# Comparison of the Efficacy of Disinfectant Pre-impregnated Wipes for Decontaminating Stainless Steel Carriers Experimentally Inoculated With Ebola Virus and Vesicular Stomatitis Virus

**DOI:** 10.3389/fpubh.2021.657443

**Published:** 2021-08-10

**Authors:** Todd A. Cutts, Samantha B. Kasloff, Jay Krishnan, Raymond W. Nims, Steven S. Theriault, Joseph R. Rubino, M. Khalid Ijaz

**Affiliations:** ^1^Canadian Science Centre for Human and Animal Health, Winnipeg, MB, Canada; ^2^J.C. Wilt Infectious Diseases Research Centre, Public Health Agency of Canada, Winnipeg, MB, Canada; ^3^RMC Pharmaceutical Solutions, Inc., Longmont, CO, United States; ^4^Department of Microbiology, The University of Manitoba, Winnipeg, MB, Canada; ^5^Reckitt Benckiser LLC, Global Research & Development for Lysol and Dettol, Montvale, NJ, United States; ^6^Department of Biology, Medgar Evers College of the City University of New York (CUNY), Brooklyn, NY, United States

**Keywords:** ASTM E2967-15, disinfectant pre-impregnated wipes, Ebola virus Makona variant, viral removal/inactivation, vesicular stomatitis virus, wiperator

## Abstract

The authors evaluated four disinfectant pre-impregnated wipes (DPW) for efficacy against Ebola virus Makona variant (EBOV) and vesicular stomatitis virus (VSV), Indiana serotype. Steel carriers were inoculated with the infectious virus and then were wiped with DPW in the Wiperator instrument per ASTM E2967-15. Following the use of J-Cloth impregnated with medium (negative control wipes) or the use of activated hydrogen peroxide (AHP)-, ethanol-, sodium hypochlorite (NaOCl)-, or single or dual quaternary ammonium compound (QAC)-based DPW, virus recovery from the carriers was assayed by titration assay and by two passages on Vero E6 cells in 6-well plates. The Wiperator also enabled the measurement of potential transfer of the virus from the inoculated carrier to a secondary carrier by the DPW or control wipes. The J-Cloth wipes wetted with medium alone (no microbicidal active) removed 1.9–3.5 log_10_ of virus from inoculated carriers but transferred ~4 log_10_ of the wiped virus to secondary carriers. DPW containing AHP, ethanol, NaOCl, or single or dual QAC as active microbicidal ingredients removed/inactivated ~6 log_10_ of the virus, with minimal EBOV or no VSV virus transfer to a secondary surface observed. In Ebola virus outbreaks, a DPW with demonstrated virucidal efficacy, used as directed, may help to mitigate the unintended spread of the infectious virus while performing surface cleaning.

## Introduction

Disinfectant pre-impregnated wipes (DPW) are an optional intervention for disrupting the cycle of infection transmission since these wipes may remove and inactivate pathogens from contaminated high-touch environmental surfaces (HITES) during use (1). Sattar and Maillard (2) correctly used the term decontamination to encompass both the physical removal and the inactivation functions of such wipes. As it is not always easy to dissect out the contributions of these two functions, decontamination seems an apt term for quantifying the net decrease in the number of infectious viruses on a cleaned surface following the use of such a wipe. As demonstrated previously (2–7), a wipe that is not impregnated with an effective disinfectant may provide physical removal only. Such a wipe, once used, may spread pathogens from a wiped contaminated surface to a secondary non-contaminated surface. For this reason, wipes that do not contain a microbicidal activity with sufficient efficacy against the target pathogen should not represent an effective intervention for limiting the spread of an infectious agent caused by indirect transmission from contaminated HITES.

We have characterized the removal, transfer, and inactivation of infectious Ebola virus Makona variant (EBOV) or vesicular stomatitis virus (VSV) by a control wipe and two DPW wipes from stainless steel carriers experimentally contaminated with the viruses in a previous study (7). Our intent in that study was to quantify the potential for transfer of infectious virus from one surface to another through the use of cleaning wipes that have no microbicidal activity (negative control wipe) or two DPW having activities with varying efficacy for the target virus. In that study, we made no conclusions regarding the relative efficacy of the two DPW for inactivating EBOV or VSV. The potential transfer of virus from a primary wiped surface to a secondary wiped surface as a result of incomplete inactivation of a virus absorbed onto a cleaning wipe is of concern, as this may represent a source of ongoing transmission of that virus.

In this study, we have used the standard testing method, ASTM E2967-15 (8), involving the Wiperator device (8–10), to evaluate the comparative efficacy of wipes containing no microbicidal active and wipes impregnated with a variety of disinfectants for decontamination of steel carriers (prototypic HITES) experimentally contaminated with a virus. We used, as challenge viruses, fully pathogenic EBOV or VSV. VSV was included, as this virus has been used previously as a surrogate for the Ebola virus [e.g., (11)]. Both are enveloped viruses. Ebola virus is a large (80 × 14,000 nm), cylindrical, negative single-stranded RNA virus of the *Filoviridae* family (12). VSV is also a large (70 × 170 nm), bullet-shaped, negative single-stranded RNA virus (13). The experimental design of the studies performed went beyond the ASTM method to include the passage of undiluted post-neutralization samples in 6-well plates of Vero E6 cells. This additional step was expected to increase the overall sensitivity of the assay for detecting any residual infectious virus following wiping.

## Materials and Methods

### Cell Line, Viruses, and Medium

African green monkey Vero E6 cells (ATCC CRL-1586; American Type Culture Collection, Manassas, VA, US) were incubated at 37°C/5% CO_2_ in DMEM (HyClone, Logan, UT, US) containing 10% fetal bovine serum (FBS; Gibco, Grand Island, NY, United States) and 10 units/mL penicillin/streptomycin (Gibco). EBOV (Ebola virus/*H. sapiens*-tc/GIN/2014/Makona-C05; GenBank accession no. KJ660348) was obtained from a clinical isolate. The virus was subsequently engineered to express a green fluorescent protein (GFP). A stock of VSV, Indiana serotype, was prepared from a reverse genetics construct (14, 15) engineered to express GFP. The virus stocks were prepared by infecting Vero E6 cells, as described in Cutts et al. (7), and were titered on the basis of the viral cytopathic effect (CPE). Titers of the stocks were determined by the Reed-Muench procedure (16) to be ≥8.8 log_10_ TCID_50_/ml.

### Negative Control J-Cloth Wipes

The negative control wipe used in this study was the “J-Cloth,” a representative material composed of cellulosic fibers from wood pulp that has been used earlier in studies of this type (8, 10). The J-Cloth contained no microbicidal activity. The sterile J-Cloth (4 × 4 cm) wipes were prepared as described previously (7).

### Preparation of DPW for Viral Decontamination Efficacy Studies

“AHP wipes” were prepared by impregnating sterile J-Cloth wipes with a 1:40 solution of accelerated hydrogen peroxide (AHP; PreEmpt, Virox Technologies, Inc., Oakville, ON, Canada), as described previously (7). “Single QAC wipes” consisted of a ready-to-use commercially available wipe composed of cellulosic pulp and polypropylene impregnated with a quaternary ammonium compound (QAC: benzyl-C12-16-alkyldimethyl chloride; Lysol Wipes; Reckitt Benckiser LLC, Montvale, NJ, United States). The three single QAC wipe lots were tested near the end of their stated expiry dates. “Dual QAC wipes” consisted of J-Cloth squares impregnated with a 1:20 solution of MicroChem (National Chemical Laboratories, Philadelphia, PA, US; lot c22c1, other lot numbers not recorded) in hard water. “Ethanol wipes” consisted of J-Cloth squares impregnated with a 66.5% ethanol solution (Commercial Alcohols, Toronto, ON, Canada; lots 029073, 026796, and 028309) in hard water. “NaOCl wipes” consisted of J-Cloth squares impregnated with a 1% NaOCl solution (Imperial Hand Sanitizer; IMP750-1; Winnipeg, MB, Canada; lot numbers not recorded). Neutralization of the disinfectants following the use of DPW was performed using neutralizers qualified for use as described in Supplementary Materials.

### Decontamination Efficacy Testing of Wipes

The testing of wipes for the ability to decontaminate a prototype environmental surface (stainless steel carriers) was performed as per ASTM 2967-15 (8). EBOV and VSV inocula were prepared in a tripartite soil load (17, 18). In the standard, the term “soil load” is intended to denote a matrix used to challenge the inactivation/removal (decontamination) of the test virus. The term “organic load” is considered equivalent to “soil load,” although the former term is more descriptive of the typical challenge matrix (secretions/excretions within which the virus is released from an infected person). The tripartite soil load consisted of sterile components (12.5 μl of 5% bovine serum albumin + 17.5 μl 5% tryptone + 50 μl 0.4% mucin) added to 170 μl of virus stock. This soil load/virus mixture was prepared fresh daily for each test replicate performed. Using a positive displacement pipette, 10 μl of virus inoculum was deposited onto sterile carriers and air-dried for 60 min in a biological safety cabinet within a BSL-2 (VSV) or BSL-4 (EBOV) laboratory prior to Wiperator (Filtaflex, Almonte, ON, Canada) testing.

**Figure 1:**
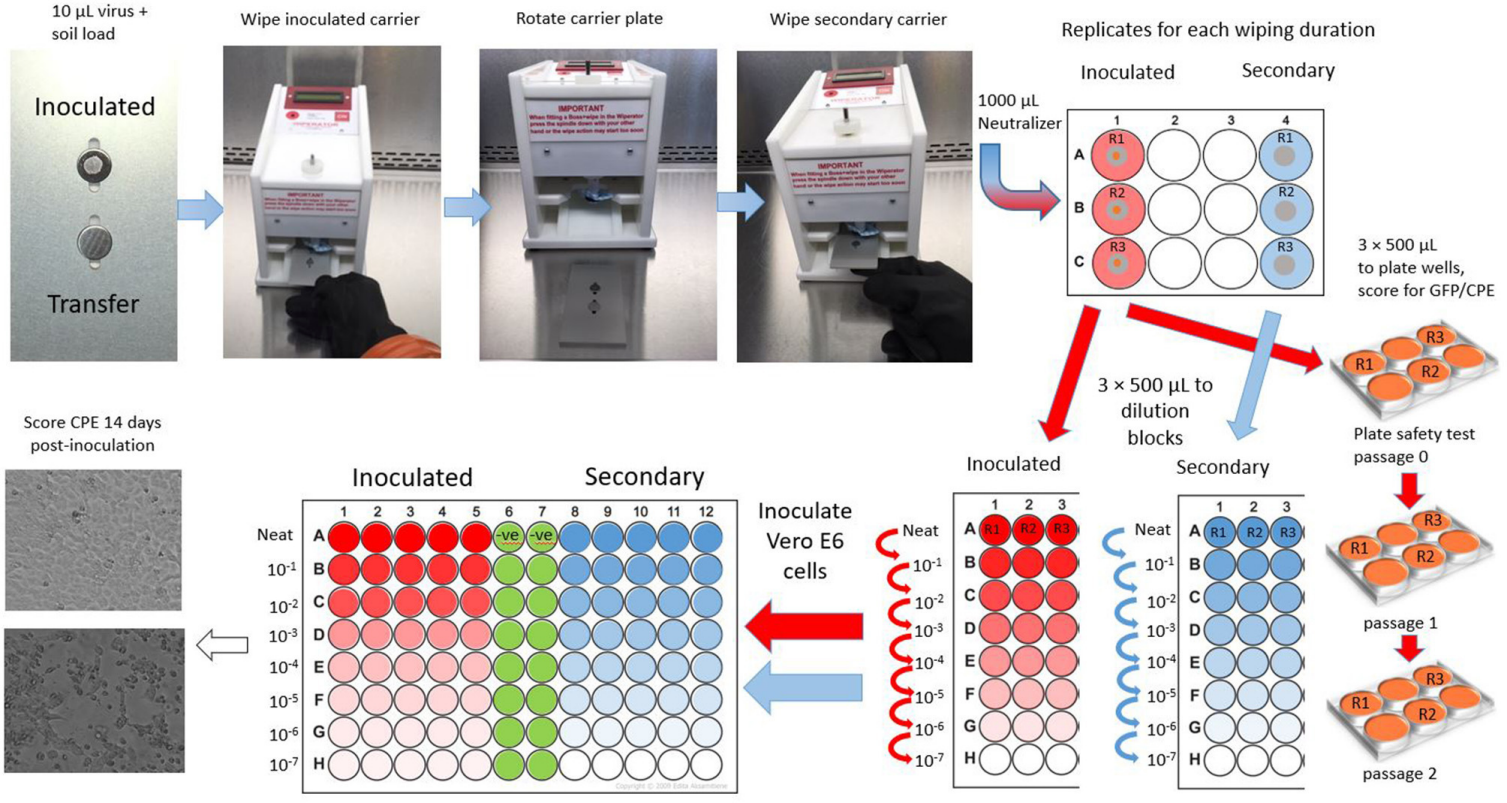
Schematic representation of the inactivation/removal testing methodology employed.

Virus-inoculated carriers were placed into a slot on one side of the Wiperator carrier plate (Figure 1, see also Supplementary Figure 1) and were held in place by a magnet on the underside of the plate. A second, non-inoculated, carrier (secondary container) was placed in a second fitted slot on the other side of the carrier plate. Sterile DPW or DMEM-impregnated wipes were removed from the Petri dishes using sterile forceps, loaded onto the Wiperator Boss (Supplementary Figure 1), and held in place with a large O-ring. The loaded Bosses were secured to the Wiperator spindle, and the plates containing the carriers were moved into place. The wiping action was programmed to start as soon as contact was made with the plate. The orbital wiping parameters were 10 mm in diameter, with 150 g of pressure, for 5-, 15-, 30-, or 60-s wiping. The 60-s wiping time, while impractical from an actual cleaning use point of view, allowed us to fully explore the inactivation time kinetics for each DPW. After being wiped, carrier plates were rotated and returned into place, exposing the uninoculated secondary carrier to the used wipe. As before, the wiping started as soon as contact was made with the carrier and continued for 5 s/cycle at 150 g pressure. Carriers were subsequently removed and aseptically transferred into 1 ml of VCM neutralizing solution (DMEM + 2% fetal calf serum + 10 units/mL penicillin/streptomycin). The presence of an infectious virus in an eluted medium from neutralized carriers was quantified by TCID_50_ assay in a 96-well plate format on Vero E6 cells (see Supplementary Materials for the procedure used). The limit of detection of the titration method for each virus was 1.3 log_10_ TCID_50_/mL, the minimum titer that can be calculated in the TCID_50_ assay employing five replicate wells per titration and in the absence of cytotoxic effects of the neutralized test sample. The plate safety test involved inoculation of 500 μL of undiluted eluate from carriers into 6-well plates of Vero E6 cells. These were monitored for CPE and GFP over 14 days (EBOV) and 4 days (VSV), after which the culture supernatant was harvested and used to inoculate fresh Vero E6 cultures. This was used to ensure that a negative result in the TCID_50_ assay was truly negative. The limit of detection of the plate safety test was determined to be 1–2 TCID_50_ per inoculation (see Supplementary Materials).

The evaluation of wipes against EBOV was performed at ambient temperature in a Class II BSC in the BSL-4 laboratories of the Public Health Agency of Canada, at the Canadian Science Center for Human and Animal Health, Winnipeg, MB, which is operated by the Government of Canada. Evaluations for VSV were conducted in a Class II BSC in the JC Wilt Infectious Disease Laboratory.

## Results

### Disinfectant Neutralizer Effectiveness Testing

The selection of the appropriate neutralizing agent for each disinfectant was informed by the results of testing described in Supplementary Materials.

### Disinfectant Efficacy Testing

The results obtained during carrier testing of the control wipes and five different DPW are displayed in Tables 1, 2 for EBOV and Tables 3, 4 for VSV, respectively. Decontamination of the inoculated carriers, representing a combination of removal and inactivation, was calculated as the difference in titer of the virus recovered from the dried untreated control carrier and of that recovered from the inoculated carrier following wiping.

**Table 1:** Efficacy of disinfectant-impregnated wipes (DPW) vs. DMEM-impregnated J-Cloth wipes for removal/inactivation (decontamination) or transfer of EBOV^*^.

**Condition**	**Untreated controls**		**EBOV titer (log**_****10****_ **TCID**_****50****_ **/mL) after wiping time:**			
	**Initial**	**Dried**	**5 s**	**15 s**	**30 s**	**60 s**
**J-Cloth DMEM Wipes**						
Inoculated carrier	6.75 ± 0.25	6.55 ± 0.28	4.09 ± 0.38	3.52 ± 0.80	3.17 ± 0.56	3.09 ± 0.69
Decontamination	–	–	2.5 log_10_	3.0 log_10_	3.4 log_10_	3.5 log_10_
Secondary carrier	–	–	3.94 ± 0.85	3.49 ± 0.63	2.91 ± 0.96	2.26 ± 0.70
**AHP DPW**						
Inoculated carrier	6.80 ± 0	6.62 ± 0.30	1.14 ± 1.56	0.32 ± 0.66	0.51 ± 1.05	0.20 ± 0.60
Decontamination	–	–	5.5 log_10_	6.3 log_10_	6.1 log_10_	6.4 log_10_
Secondary carrier	–	–	0.93 ± 1.40	ND	0.12 ± 0.35	ND
**Single QAC DPW**						
Inoculated carrier	6.90 ± 0.15	6.59 ± 0.27	0.58 ± 0.93	ND	0.19 ± 0.56	ND
Decontamination	–	–	6.0 log_10_	6.6 log_10_	6.4 log_10_	6.6 log_10_
Secondary carrier	–	–	0.20 ± 0.60	ND	ND	ND
**Dual QAC DPW**						
Inoculated carrier	6.98 ± 0.72	6.00 ± 071	ND	ND	ND	ND
Decontamination	–	–	6.0 log_10_	6.0 log_10_	6.0 log_10_	6.0 log_10_
Secondary carrier	–	–	ND	ND	ND	ND
**Ethanol DPW**						
Inoculated carrier	6.82 ± 0.18	6.64 ± 0.49	ND	ND	ND	ND
Decontamination	–	–	6.6 log_10_	6.6 log_10_	6.6 log_10_	6.6 log_10_
Secondary carrier	–	–	ND	ND	ND	ND
**NaOCl DPW**						
Inoculated carrier	6.13	6.34 ± 0.16	ND	ND	ND	ND
Decontamination	–	–	6.3 log_10_	6.3 log_10_	6.3 log_10_	6.3 log_10_
Secondary carrier	–	–	ND	ND	ND	ND

*Values displayed are the log_10_ infectious virus titer in units of log_10_ TCID_50_/mL for untreated controls and the results of Wiperator post-testing after various wiping times. The results are the mean ± SD for n = 9 [three replicates each in each of the three assays]. ND, not detected. “Decontamination” values shown for the J-Cloth wipe represent the mean log_10_ of the infectious virus load (TCID_50_) for the dried untreated control carrier minus the mean log_10_ of the infectious virus recovered from the inoculated carrier. “Transfer” is expressed as the percent of the infectious virus recovered from the dried untreated control carrier that is recovered from the secondary carrier. Note: the data reported for J-Cloth DMEM Wipes, AHP DPW, and single QAC DPW were previously published in the modified form (7).

**Table 2:** Plate safety test results for inactivation of EBOV by DPW vs. DMEM-impregnated J-Cloth wipes^*^.

	**Plate safety test result (presence of GFP)**		
**Test condition**	**Trial 1**	**Trial 2**	**Trial 3**
**(contact time)**			
**AHP DPW**			
Negative control	**-, -, -**	**-, -, -**	**-, -, -**
Wipe + Neutralizer	NT	NT	NT
5 s	+, +, +	**-**, +, **-**	**-**, +, **-**
15 s	+, +, +	**-**, +, +	**-, -**, +
30 s	+, +, **-**	**-, -, -**	**-**, +,**-**
60 s	**-, -, -**	**-, -, -**	+, **-, -**
**Single QAC DPW**			
Negative control	**-, -, -**	**-, -, -**	**-, -, -**
Wipe + Neutralizer	**-, -, -**	**-, -, -**	**-, -, -**
5 s	+, +, +	**-**, +, **-**	**-, -, -**
15 s	**-, -, -**	**-, -, -**	**-, -, -**
30 s	**-, -, -**	**-, -, -**	**-**, +, **-**
60 s	**-, -, -**	**-, -, -**	**-, -, -**
**Dual QAC DPW**			
Negative control	**-, -, -**	**-, -, -**	**-, -, -**
Wipe + Neutralizer	**-, -, -**	**-, -, -**	**-, -, -**
5 s	**-, -, -**	**-, -, -**	**-, -, -**
15 s	**-, -, -**	**-, -, -**	**-, -, -**
30 s	**-, -, -**	**-, -, -**	**-, -, -**
60 s	**-, -, -**	**-, -, -**	**-, -, -**
**Ethanol DPW**			
Negative control	**-, -, -**	**-, -, -**	**-, -, -**
Wipe + Neutralizer	**-, -, -**	**-, -, -**	**-, -, -**
5 s	**-, -, -**	**-, -, -**	**-, -, -**
15 s	**-, -, -**	**-, -, -**	**-, -, -**
30 s	**-, -, -**	**-, -, -**	**-, -, -**
60 s	**-, -, -**	**-, -, -**	**-, -, -**
**NaOCl Wipes**			
Negative control	**-, -, -**	**-, -, -**	**-, -, -**
Wipe + Neutralizer	**-, -, -**	**-, -, -**	**-, -, -**
5 s	**-**, +, **-**	**-**, +, **-**	**-, -, -**
15 s	**-, -, -**	**-, -, -**	**-**, +, **-**
30 s	**-, -, -**	**-, -, -**	**-, -, -**
60 s	**-, -, -**	**-, -, -**	**-, -, -**

*The Vero E6 cultures were inoculated with 500 μL of undiluted neutralized samples from the inoculated carriers and passaged two times. Disinfectant pre-impregnated wipes; +, viral green fluorescent protein (GFP) observed; –, viral GFP not observed, or no cytotoxicity observed for N + wipes (neutralizer + wipes cytotoxicity control). NT, not tested.

**Table 3:** Efficacy of DPW vs. DMEM-impregnated J-Cloth wipes for removal/inactivation (decontamination) or transfer of vesicular stomatitis virus (VSV)^*^.

**Condition**	**Untreated controls**		**VSV titer (log**_****10****_ **TCID**_****50****_ **/mL) after wiping time:**			
	**Initial**	**Dried**	**5 s**	**15 s**	**30 s**	**60 s**
**J-Cloth DMEM Wipes**						
Inoculated carrier	6.80 ± 0.00	5.78 ± 0.33	3.85 ± 0.51	3.40 ± 0.41	3.46 ± 0.36	3.32 ± 0.40
Decontamination	–	–	1.9 log_10_	2.4 log_10_	2.3 log_10_	2.5 log_10_
Secondary carrier	–	–	3.79 ± 0.57	3.48 ± 0.39	3.26 ± 0.58	3.36 ± 0.34
**AHP DPW**						
Inoculated carrier	7.43 ± 0.71	6.21 ± 1.07	ND	ND	ND	ND
Decontamination	–	–	6.2 log_10_	6.2 log_10_	6.2 log_10_	6.2 log_10_
Secondary carrier	–	–	ND	ND	ND	ND
**Single QAC DPW**						
Inoculated carrier	6.47	6.02 ± 0.19	ND	ND	ND	ND
Decontamination	–	–	6.0 log_10_	6.0 log_10_	6.0 log_10_	6.0 log_10_
Secondary carrier	–	–	ND	ND	ND	ND
**Dual QAC DPW**						
Inoculated carrier	6.98 ± 0.72	6.00 ± 0.71	ND	ND	ND	ND
Decontamination	–	–	6.0 log_10_	6.0 log_10_	6.0 log_10_	6.0 log_10_
Secondary carrier	–	–	ND	ND	ND	ND
**Ethanol DPW**						
Inoculated carrier	6.73 ± 0.07	5.48 ± 0.33	0.90 ± 0.97	ND	ND	ND
Decontamination	–	–	4.6 log_10_	5.5 log_10_	5.5 log_10_	5.5 log_10_
Secondary carrier	–	–	ND	ND	ND	ND
**NaOCl DPW**						
Inoculated carrier	6.93	5.48 ± 0.32	0.20 ± 0.60	ND	ND	ND
Decontamination	–	–	5.3 log_10_	5.5 log_10_	5.5 log_10_	5.5 log_10_
Secondary carrier	–	–	ND	ND	ND	ND

*Values displayed are the log_10_ infectious virus titer in units of log_10_ TCID_50_/mL for untreated controls and the results of Wiperator post-testing after various wiping times. The results are the mean ± SD for n = 9 [three replicates each in each of the three assays]. ND, not detected. “Decontamination” values shown for the J-Cloth wipe represent the mean log_10_ of the infectious virus load (TCID_50_) for the dried untreated control carrier minus the mean log_10_ of the infectious virus recovered from the inoculated carrier. “Transfer” is expressed as the percent of the infectious virus recovered from the dried untreated control carrier that is recovered from the secondary carrier. Note: the data reported for J-Cloth DMEM Wipes, AHP DPW, and single QAC DPW were previously published in modified form (7).

**Table 4:** Plate safety test results for the inactivation of VSV by DPW vs. DMEM-impregnated J-Cloth wipes^*^.

	**Plate safety test result (presence of GFP)**		
**Test condition**	**Trial 1**	**Trial 2**	**Trial 3**
**(contact time)**			
**AHP DPW**			
Negative control	**-, -, -**	**-, -, -**	**-, -, -**
Wipe + Neutralizer	**-, -, -**	**-, -, -**	**-, -, -**
5 s	**-, -, -**	**-, -, -**	**-, -, -**
15 s	**-, -, -**	**-, -, -**	**-, -, -**
30 s	**-, -, -**	**-, -, -**	**-, -, -**
60 s	**-, -, -**	**-, -, -**	**-, -, -**
**Single QAC DPW**			
Negative control	**-, -, -**	**-, -, -**	**-, -, -**
Wipe + Neutralizer	**-, -, -**	**-, -, -**	**-, -, -**
5 s	**-, -, -**	**-, -, -**	**-, -, -**
15 s	**-, -, -**	**-, -, -**	**-, -, -**
30 s	**-, -, -**	**-, -, -**	**-, -, -**
60 s	**-, -, -**	**-, -, -**	**-, -, -**
**Dual QAC DPW**			
Negative control	**-, -, -**	**-, -, -**	**-, -, -**
Wipe + Neutralizer	**-, -, -**	**-, -, -**	**-, -, -**
5 s	**-, -, -**	**-, -, -**	**-, -, -**
15 s	**-, -, -**	**-, -, -**	**-, -, -**
30 s	**-, -, -**	**-, -, -**	**-, -, -**
60 s	**-, -, -**	**-, -, -**	**-, -, -**
**Ethanol DPW**			
Negative control	**-, -, -**	**-, -, -**	**-, -, -**
Wipe + Neutralizer	**-, -, -**	**-, -, -**	**-, -, -**
5 s	+, +, +	**-, -**, +	**-, -, -**
15 s	**-, -, -**	**-, -, -**	**-, -, -**
30 s	**-, -, -**	**-, -, -**	**-, -, -**
60 s	**-, -, -**	**-, -, -**	**-, -, -**
**NaOCl DPW**			
Negative control	**-, -, -**	**-, -, -**	**-, -, -**
Wipe + Neutralizer	**-, -, -**	**-, -, -**	**-, -, -**
5 s	**-, -, -**	**-, -, -**	-, +, -
15 s	**-, -, -**	**-, -, -**	**-, -, -**
30 s	**-, -, -**	**-, -, -**	**-, -, -**
60 s	**-, -, -**	**-, -, -**	**-, -, -**

*The Vero E6 cultures were inoculated with 500 μL of undiluted neutralized samples from the inoculated carriers and passaged twice. +, Viral GFP observed; –, viral GFP not observed, or no cytotoxicity observed for N + wipes (neutralizer + wipes cytotoxicity control).

In the absence of expected viral inactivation, as in the case of the control wipe, decontamination primarily represents “removal.” The extent of virus “transfer” is expressed as a percentage of the virus on the inoculated carrier that was subsequently recovered from the secondary carrier after the 5-s transfer wiping step. “Inactivation,” in the case of DPW, may be implied, but not quantified, by the reduction of viral recovery from the inoculated carriers following wiping for 5, 15, 30, or 60 s and of viral recovery from the secondary carrier following the 5-s transfer wiping step.

The amount of infectious virus remaining on the wipes themselves was not measured. Complete mass balances for the spiking viruses were, therefore, not obtained. The results of the Wiperator study performed to evaluate the efficacy of DPW for decontaminating EBOV-inoculated carriers and VSV-inoculated carriers are displayed in Tables 1, 3, respectively. The values shown represent the combined data from three trials (three replicates each) utilizing one lot of the AHP wipe and one trial each (three replicates per trial) for three lots of the single QAC wipe and dual QAC, ethanol, and NaOCl wipes. The results of the plate safety test for residual infectious EBOV and VSV are displayed in Tables 2, 4, respectively.

### Results for EBOV

#### Negative Control Wipes

Ebola virus Makona variant titers recovered from the carriers after use of the DMEM-impregnated J-Cloth (control wipe) were 3.1– 4.1 log_10_ TCID_50_/mL for the 5-, 15-, 30-, and 60-s wiping times, compared with an initial titer of 6.6 log_10_ TCID_50_/mL. The log_10_ decontamination of EBOV from the inoculated carriers for the various wiping times ranged from 2.5 to 3.5 log_10_, with minimal increases in the extent of decontamination observed with increasing wiping time (Table 1). In the transfer step, the EBOV titers recovered from the secondary carriers were 2.3–3.9 log_10_ TCID_50_/mL following transfer from the inoculated carriers wiped for 5, 15, 30, or 60 s. These results indicate that the J-Cloth wipes impregnated with DMEM removed EBOV from the original contaminated surface while transferring a portion of the infectious virus to the secondary surface.

#### AHP DPW

Ebola virus Makona variant titers recovered from the inoculated carriers after use of the AHP DPW ranged from 0.20 to 1.1 log_10_ TCID_50_/mL; *n* = 9 replicates/time point) (Table 1). The log_10_ decontamination of EBOV from the inoculated carriers for the various wiping times ranged from 5.5 to 6.4 log_10_. The results of the plate safety assay (Table 2) confirmed that five of nine, six of nine, three of nine, and one of nine replicates were positive for residual infectious virus for the 5-, 15-, 30-, and 60-s wiping times, respectively. The EBOV titers recovered from the secondary carriers during the transfer step were 0.9 log_10_ TCID_50_/mL and 0.1 log_10_ TCID_50_/mL (*n* = 9 replicates) following transfer from the inoculated carriers wiped for 5 and 30 s, respectively. The virus was transferred from three of the nine replicates of the inoculated carriers wiped for 5 s and from a single replicate of the inoculated carriers wiped for 30 s. No detectable infectious EBOV was transferred from the inoculated carriers wiped for 15 or 60 s.

#### Single QAC DPW

Ebola virus Makona variant (0.6 and 0.2 log_10_ TCID_50_/mL; *n* = 9 replicates) was recovered from carriers after 5-s and 30-s wiping with the single QAC DPW, respectively (Table 1). The log_10_ decontamination of EBOV from the inoculated carriers for the various wiping times ranged from 6.0 to 6.6 log_10_. After 15-s and 60-s wiping, no infectious virus was recovered from the inoculated carriers. The results of the plate safety assay (Table 2) confirmed the positive and negative results. The EBOV titers recovered on the secondary carriers following the transfer step were very low (mean 0.2 log_10_ TCID_50_/mL; *n* = 9 replicates). No infectious EBOV was recovered from the secondary carriers following the transfer from carriers wiped for 15, 30, or 60 s.

#### Dual QAC DPW, NaOCl DPW, and Ethanol DPW

No infectious EBOV was recovered from carriers after use of the Dual QAC, NaOCl, or ethanol DPW, evaluated after 5−60-s wiping (Table 1). The decontamination of EBOV from the inoculated carriers for the various wiping times ranged from 6.0 to 6.6 log_10_. The results of the plate safety assay (Table 2) confirmed the absence of infectious virus in these replicates for the dual QAC and ethanol DPW. For the NaOCl DPW, the virus was recovered from two replicates at 5 s and one replicate at 15 s. No infectious EBOV was recovered from secondary carriers during the transfer step from the inoculated carriers wiped for 5, 15, 30, or 60 s.

### Results for VSV

#### Negative Control Wipes

Vesicular stomatitis virus titers recovered from the carriers following wiping with the DMEM-impregnated J-Cloth were 3.3 to 3.9 log_10_ TCID_50_/mL for the 5-, 15-, 30-, and 60-s wiping times, compared with an initial titer of 5.8 log_10_ TCID_50_/mL. The log_10_ decontamination of VSV from the inoculated carriers for the various wiping times ranged from 1.9 to 2.5 log_10_, with minimal increases in the extent of decontamination observed with an increase in the wiping time (Table 3). In the transfer step, the VSV titers recovered from the secondary carriers were 3.3 to 3.8 log_10_ TCID_50_/mL following transfer from the inoculated carriers wiped for 5, 15, 30, or 60 s. These results indicate that the J-Cloth wipes impregnated with DMEM removed VSV from the original contaminated surface while transferring a portion of the infectious virus to the secondary surface.

#### NaOCl DPW

Vesicular stomatitis virus (mean titer 0.20 log_10_ TCID_50_/mL; *n* = 9 replicates) was recovered after use of the NaOCl DPW from one of nine replicates evaluated after 5-s wiping (Table 3), indicating incomplete (5.3 log_10)_ decontamination for this condition. This result was confirmed in the plate safety test (Table 4). No infectious VSV was recovered from secondary containers during the transfer step from the inoculated carriers wiped for 5, 15, 30, or 60 s.

#### Ethanol DPW

Vesicular stomatitis virus (mean titer 0.9 log_10_ TCID_50_/mL; *n* = 9 replicates) was recovered after use of the ethanol DPW from carriers evaluated after 5-s wiping (Table 3). The log_10_ decontamination of VSV from the inoculated carriers for the various wiping times ranged from 4.6 to 5.5 log_10_. The results of the plate safety assay (Table 4) confirmed that four of the nine replicates were positive for residual infectious virus for the 5-s wiping time. No infectious virus was recovered from the inoculated carriers wiped for 15, 30, and 60 s. No infectious VSV was recovered from secondary containers during the transfer step from the inoculated carriers wiped for 5, 15, 30, or 60 s.

#### AHP DPW, Single QAC DPW, and Dual QAC-DPW

For each of these three DPW, VSV was not able to be recovered from the inoculated carriers after the 5-, 15-, 30-, or 60-s wiping times (Table 3). These results were confirmed by the results of the plate safety test (Table 4). This indicates that these DPW removed or inactivated essentially all of the VSV deposited on the original contaminated surface (6.0 to 6.2 log_10_ TCID_50_/mL, estimated on the basis of the value for the dried untreated control carrier). No infectious VSV was recovered from the secondary carriers following transfer from the inoculated carriers wiped for 5, 15, 30, or 60 s.

## Discussion

Disposable DPW represent an option for reducing pathogen loads on HITES in healthcare and home settings (1, 19). The two orthogonal functionalities of DPW, removal and inactivation, together have been referred to as “decontamination” by Sattar and Maillard (2), and we have adopted this terminology in the present investigation. The inactivation function of a DPW is determined by the efficacy of the incorporated disinfectant against the target virus. A DPW effective for one enveloped virus should also be effective against other enveloped viruses, per the well-established hierarchy of pathogen susceptibility to microbicides (20–23). The inactivation resulting from DPW use may occur within the wipe itself or within the liquid expressed from the wipe during wiping of the original HITES or secondary HITES.

For an emerging virus such as those causing hemorrhagic fever, what level of decontamination efficacy is required? For such viruses, a low level of virus transferred from a contaminated surface to a secondary surface may result in the spread of infection to otherwise healthy individuals coming in contact with contaminated HITES. For instance, the infectious dose for the Ebola virus has been estimated to be 1–10 infectious units (24, 25). If a wipe having no or limited microbicidal activity (such as the DMEM-impregnated J-Cloth wipe used as a negative control in this study) was to be used for cleaning, removal of EBOV would be expected, although the lack of inactivation suggests that a portion of the virus could be spread to another surface during the wiping process (7). The spread of even a low percentage of the removed virus by such a wipe (in our study the control wipe transferred >3.2 log_10_, equating to ~1500 infectious units) from the contaminated HITES to a new surface could represent a significant opportunity for transmitting infectious Ebola virus.

In the present study, we used the ASTM-2967-15 method to evaluate the efficacy of DPW containing one of five actives (AHP, Single QAC, Dual QAC, NaOCL, and ethanol) against EBOV and VSV. These enveloped viruses were expected to be readily inactivated by each of the formulated actives tested. The comparative efficacy of DPW containing different microbicidal actives for decontaminating prototypic HITES inoculated with Ebola virus or VSV has not previously been explored. Our recent article (7) addressed the risk of transfer of these viruses from the original wiped surface to a secondary surface and did not specifically address comparative decontamination efficacy. Becker et al. (6) evaluated four DPW for inactivating three non-enveloped viruses (adenovirus, simian virus 40, and murine norovirus) using the EN 16615 methodology (26). In that study, only a DPW containing a peracetic acid active was found to cause sufficient inactivation of the three viruses to prevent transfer to secondary surfaces during wiping. The DPW with QAC or 2-propanol as active ingredients were found to transfer one or more of the test viruses. This likely reflects the relatively lesser susceptibility of non-enveloped viruses to certain disinfectants (20–24, 27).

In the case of EBOV, the dual QAC DPW and ethanol DPW were the most effective DPW (complete, 6.0 log_10_ decontamination after wiping times of 5–60 s). The single QAC DPW required 60 s to cause complete decontamination (6.0 to 6.6 log_10_), while the AHP DPW failed to cause complete decontamination after wiping for up to 60 s. The recovery of infectious EBOV following 30-s wiping with the single QAC DPW, but not following 15-s wiping, was unexpected. This may represent sampling issues (i.e., Poisson distribution) as the titers of the virus approached the limit of detection.

The rhabdovirus VSV was found to be more susceptible to decontamination by the AHP DPW and single QAC DPW than EBOV, while being less susceptible to ethanol DPW. The AHP, single QAC, and dual QAC DPW caused complete inactivation at 5−60-s wiping. The structural differences between filoviruses and rhabdoviruses may play a role in the differences in efficacy observed. For instance, filoviruses have more extended tubular structures (80 × 14,000 nm) (12) whereas rhabdoviruses are described as bullet shaped (70 × 170 nm) (13). The extended tubular shape may predispose the filovirus to interact with the cell membranes in the virus stock and with the components of the organic load employed as part of the challenge virus matrix. Taken together, these data suggest that, during outbreaks involving emerging/re-emerging viruses such as Ebola virus, a DPW with demonstrated virucidal efficacy, used as directed, may prevent unintended spread of the infectious virus while performing cleaning of HITES.

We acknowledge the following limitations to our study, which should be kept in mind when considering these results. First, we evaluated viruses deposited on stainless steel carriers only. There are other prototypic HITES surfaces, such as plastic, tile/ceramic, wood, or laminate counter tops, that could potentially be disinfected using DPW. It may not be appropriate to extrapolate our findings using non-porous stainless steel carriers to each of the other types of HITES surfaces. Second, we were able to evaluate wipes composed of cellulose and cellulose/polypropylene only. There may be DPW composed of other fabric types. Our results using cellulose or cellulose/polypropylene wipes should be extrapolated to DPW composed of other fabric types with caution. Finally, we evaluated two enveloped RNA viruses in this study, as our primary interest was in the Ebola virus and a surrogate, VSV. We did not challenge the DPW with an enveloped DNA virus or with any non-enveloped viruses. We expect that the DPW should have a similar efficacy for any enveloped virus on the basis of the known hierarchy of susceptibility of viruses to microbicidal active ingredients (22–24). Efficacy for the relatively less susceptible non-enveloped viruses (22–24) should not be inferred from our results.

## Conclusion

In this investigation, we have identified differences in decontamination efficacy among five DPW with differing microbicidal actives. The dual QAC DPW and ethanol DPW were the most effective for EBOV, causing 6.0 to 6.6 log_10_ inactivation. NaOCl DPW and single QAC DPW required 30 s and 60 s, respectively, to cause complete inactivation of EBOV. The AHP DPW did not cause complete decontamination of EBOV after ≤ 60-s wiping. Each of the DPW were effective against VSV, causing complete (5.5 to 6.2 log_10_) inactivation at 15-, 30-, and 60-s wiping. During outbreaks involving emerging/re-emerging viruses such as Ebola virus, a DPW with demonstrated virucidal efficacy, used as directed, may help to mitigate the unintended spread of the infectious virus while performing cleaning of HITES.

## Data Availability Statement

The original contributions presented in the study are included in the article's Supplementary Material, further inquiries can be directed to the Corresponding Author.

## Author Contributions

MI, TC, and ST conceived and designed the experiments. RN, MI, and TC analyzed the data. RN, MI, JR, TC, ST, JK, and SK each contributed to the writing of the manuscript. All authors reviewed and approved the manuscript.

## Conflict of Interest

JR and MI are employed by RB, who also provided partial funding support for this study. RN provided data analysis and manuscript preparation as a paid consultant employed by RMC Pharmaceutical Solutions, Inc. The remaining authors declare that the research was conducted in the absence of any commercial or financial relationships that could be construed as a potential conflict of interest.

## Publisher's Note

All claims expressed in this article are solely those of the authors and do not necessarily represent those of their affiliated organizations, or those of the publisher, the editors and the reviewers. Any product that may be evaluated in this article, or claim that may be made by its manufacturer, is not guaranteed or endorsed by the publisher.

## References

[1] Lopez G Kitajima M Havas A Gerba C Reynolds K. Evaluation of a disinfectant wipe intervention on fomite-to-finger microbial transfer. Appl Environ Microbiol. (2014) 80:3113–8. 10.1128/AEM.04235-13 24610856 PMC4018905

[2] Sattar S Maillard J. The crucial role of wiping in decontamination of high-touch environmental surfaces: review of current status and directions for the future. Am J Infect Control. (2013) 41:S97–104. 10.1016/j.ajic.2012.10.032 23622759

[3] Sattar SA. Promises and pitfalls of recent advances in chemical means of preventing the spread of nosocomial infections by environmental surfaces. Am J Infect Control. (2010) 38:S34. 10.1016/j.ajic.2010.04.207 20569854

[4] Wesgate R Maillard J. Efficacy of commercially available disinfectant wipes to remove, transfer and kill a surrogate human viral pathogen *in vitro*. Presented at the UK Healthcare Infection Society, in association with La Société Française d'Hygiène Hospitalière. (2014). Available online at: https://www.trycare.co.uk/files/ww/Cardiff%20University%20-%20Efficacy%20of%20disinfectant%20wipes.pdf

[5] Lei H Li Y Xiao S Yang X Lin C Norris S. Logistic growth of a surface contamination network and its role in disease spread. Sci Rpt. (2017) 7:14826. 10.1038/s41598-017-13840-z 29093534 PMC5665872

[6] Becker B Henningsen L Paulmann D Bischoff B Todt D Steinmann E. Evaluation of the virucidal efficacy of disinfectant wipes with a test method simulating practical conditions. Antimicrob Resist Infect Control. (2019) 8:121. 10.1186/s13756-019-0569-4 31346462 PMC6636036

[7] Cutts T Robertson C Theriault S Nims R Kasloff S Rubino J. Assessing the contributions of inactivation, removal, and transfer of Ebola virus and vesicular stomatitis virus by disinfectant pre-soaked wipes. Front Pub Health. (2020) 8:183. 10.3389/fpubh.2020.00183 32582604 PMC7280553

[8] ASTM International. ASTM E2967-15. Standard Test Method for Assessing the Ability of Pre-wetted Towelettes to Remove and Transfer Bacterial Contamination on Hard, Non-Porous Environmental Surfaces Using the Wiperator. West Conshohocken, PA: ASTM International (2015). Available online at: https://webstore.ansi.org/standards/astm/astme296715

[9] Ramm L Siani H Wesgate R Maillard J. Pathogen transfer and high variability in pathogen removal by detergent wipes. Am J Infect Control. (2015) 43:724–8. 10.1016/j.ajic.2015.03.024 25997876

[10] Sattar S Bradley C Kibbee R Wesgate R Wilkinson M Sharpe T. Disinfectant wipes are appropriate to control microbial bioburden from surfaces: use of a new ASTM standard test protocol to demonstrate efficacy. J Hosp Infect. (2015) 91:319–25. 10.1016/j.jhin.2015.08.026 26518272

[11] Nikiforuk A Cutts T Theriault S Cook BW. Challenge of liquid stressed protective materials and environmental persistence of Ebola virus. Sci Rep. (2017) 7:4388 10.1038/s41598-017-04137-2 PMC5491502 28663587

[12] Salata C Calistri A Alvisi G Celestino M Parolin Palù G. Ebola virus entry: from molecular characterization to drug discovery. Viruses. (2019) 11:274. 10.3390/v11030274 30893774 PMC6466262

[13] Acha P Szyfres B. Vesicular Stomatitis. Zoonoses and Communicable Diseases Common to Man and Animals. 3rd ed. Washington D.C.:Pan American Health Organization. (2003) p. 347–55.

[14] Lawson N Stillman E Whitt M Rose J. Recombinant vesicular stomatitis viruses from DNA. Proc Natl Acad Sci USA. (1995) 92:4477–81. 10.1073/pnas.92.10.4477 7753828 PMC41967

[15] Dalton K Rose J. Vesicular stomatitis virus glycoprotein containing the entire green fluorescent protein on its cytoplasmic domain is incorporated efficiently into virus particles. Virology. (2001) 279:414–21. 10.1006/viro.2000.0736 11162797

[16] Reed L Muench H. A simple method of estimating fifty percent endpoints. Am J Hygiene. (1938) 27:493–7. 10.1093/oxfordjournals.aje.a118408

[17] Sattar S Ansari S. The fingerpad protocol to assess hygienic hand antiseptics against viruses. J Virol Meth. (2002) 103:171–81. 10.1016/S0166-0934(02)00025-3 12008011

[18] ASTM International. ASTM E1052-11. Standard Test Method to Assess the Activity of Microbicides against Viruses in Suspension. (2011). Available online at: https://www.astm.org/Standards/E1052.htm

[19] Song X Vossebein L Zille A. Efficacy of disinfectant-impregnated wipes used for surface disinfection in hospitals: a review. Antimicrob Resist Infect Control. (2019) 8:139 10.1186/s13756-019-0595-2 PMC6701098 31452873

[20] Klein M Deforest A. Principles of viral inactivation. In: Block SS, editor. Disinfection, sterilization, and preservation, 3rd edition. Philadelphia: Lea and Febiger (1983). p. 422–34.

[21] Sattar SA. Hierarchy of susceptibility of viruses to environmental surface disinfectants: a predictor of activity against new and emerging viral pathogens. J AOAC Int. (2007) 90:1655–8. 10.1093/jaoac/90.6.1655 18193744

[22] Ijaz M Rubino J. Should test methods for disinfectants use vertebrate virus dried on carriers to advance virucidal claims? Inf Control Hosp Epidemiol. (2008) 29:192–4. 10.1086/526441 18179381

[23] Ijaz M Sattar S Rubino J Nims R Gerba C. Combating SARS-CoV-2: leveraging microbicidal experiences with other emerging/re-emerging viruses. Peer J. (2020) 8:e9914. 10.7717/peerj.9914 33194365 PMC7485481

[24] Bibby K Fischer R Casson L de Carvalho N Haas C Munster V. Disinfection of Ebola virus in sterilized municipal wastewater. PLoS Negl Trop Dis. (2017) 11:e0005299. 10.1371/journal.pntd.0005299 28146555 PMC5287448

[25] Franz D Jahrling P Friedlander A McClain D Hoover D Byrne W. Clinical recognition and management of patients exposed to biological warfare agents. J Am Med Assoc. (1997) 278:399–411. 9244332 10.1001/jama.278.5.399

[26] CEN-Comité Europeen de Normalisation. EN 16615:2015 *Chemical disinfectants and antiseptics – quantitative test method for the evaluation of bactericidal and yeasticidal activity on non-porous surfaces with mechanical action employing wipes in the medical area (4-field test) – test method and requirements (phase 2, step 2)*. (2015). Available online at: https://infostore.saiglobal.com/en-us/Standards/EN-16615-2015-340824_SAIG_CEN_CEN_780961/

[27] International Conference on Harmonisation. ICH Topic Q5A(R1). Viral safety evaluation of biotechnology products derived from cell lines of human or animal origin. (1999). Available online at: https://www.ema.europa.eu/en/ich-q5a-r1-quality-biotechnological-products-viral-safety-evaluation-biotechnology-products-derived

