# Supplementary material for: Comparison of the Efficacy of Disinfectant Pre-impregnated Wipes for Decontaminating Stainless Steel Carriers Experimentally Inoculated With Ebola Virus and Vesicular Stomatitis Virus
Source: Front Public Health. 2021 Aug 10;9:657443. doi: 10.3389/fpubh.2021.657443 (PMC8383043; doi:10.3389/fpubh.2021.657443)
Supplement: Supplementary file 1 [file Data_Sheet_1.PDF]

Supplementary Materials for

Comparison of the Efficacy of Disinfectant Pre-Impregnated Wipes for Decontaminating Stainless Steel Carriers Experimentally Inoculated with Ebola Virus and Vesicular Stomatitis Virus

Todd A. Cutts[1,2], Samantha B. Kasloff[1,2], Jay Krishnan[1,2], Raymond W. Nims[3], Steven S. Theriault[4], Joseph R. Rubino[5], M. Khalid Ijaz[5,6]*

[1] *Canadian Science Centre for Human and Animal Health, Winnipeg, MB, Canada,* [2] *J.C. Wilt Infectious Diseases Research Centre, Public Health Agency of Canada, Winnipeg, MB, Canada,* [3] *Department of Microbiology, The University of Manitoba, Winnipeg, MB, Canada,* [4] *RMC Pharmaceutical Solutions, Inc., Longmont, CO, United States,* [5] *Reckitt Benckiser LLC, Global Research & Development for Lysol and Dettol, Montvale, NJ, United States,* [6] *Department of Biology, Medgar Evers College of the City University of New York (CUNY), Brooklyn, NY, United States*

Wiperator

The major components of the Wiperator device are shown in Supplementary Figure S1.

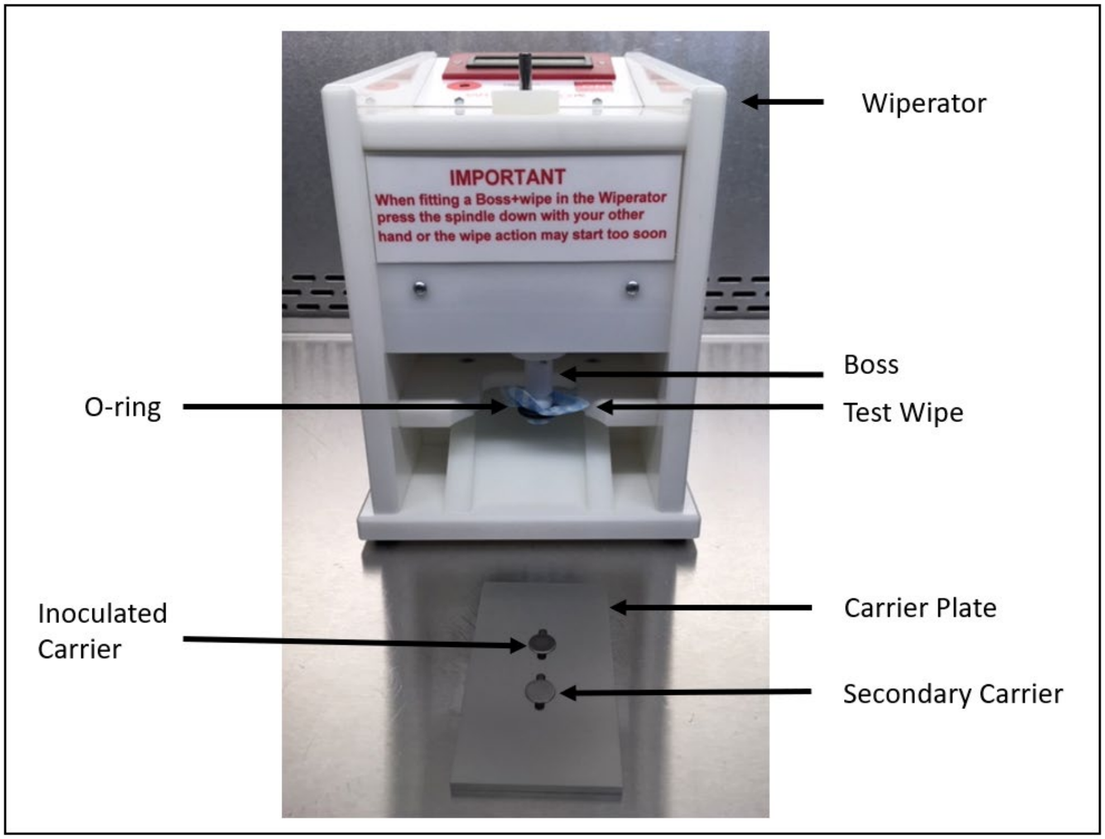

Supplementary Figure S1. Components of the Wiperator used to investigate removal, transfer, and inactivation of VSV and EBOV/Mak by DPW and control wipes (**7**).

$TCID_{50}$ titration method

Vero E6 cells were seeded on the day prior to use in the titration assay in a 96-well plate format to attain 80% confluence on the day of testing. Virus stocks, or virus dried in the presence of a soil load, treated, and eluted into 1ml of virus culture medium (VCM) by repeat pipetting, were ten-fold serially diluted in VCM. In replicates of 5 per dilution series, 50 µl of diluted virus was added to wells of Vero E6 cells containing 150 µL of VCM. The inoculated 96-well plates were then incubated at 37°C +5% CO_2 for 14 days (EBOV) or 4 days (VSV). Plates were examined for CPE and GFP during this time period using a light microscope and fluorescent microscope, respectively, and compared to a negative control to determine viral titer in $TCID_{50}$ by the Reed Muench procedure [reference provided in Main Text].

Method for disinfectant neutralization efficacy testing

Prior to evaluating the efficacy of wipes for decontaminating infectious virus (Ebola virus or VSV), a neutralization assay was performed to evaluate the interaction of neutralizers and disinfectants, cytotoxicity to the Vero E6 cells used for reporting GFP/CPE, and impacts on the recovery of infectious virus from the surfaces post-test. Combinations of neutralizers, disinfectants, and viruses were evaluated.

Several neutralizing reagents (VCM, 1x Letheen Broth, and 1× Letheen Broth + VCM [1:10 10× Letheen broth stock combined with VCM]) were prepared fresh on the day of use. In addition, a low titer virus stock (~100-1000 $TCID_{50}$ per mL) of VSV or EBOV/Mak was also prepared with only 10 µl inoculum used for the appropriate test conditions requiring live virus. All neutralization controls were performed in replicates of three over a single experiment.

Neutralization controls included the following:

Negative Control: Cells were cultured in VCM and used as a control for evaluation of cytotoxicity and viral CPE.

Neutralizer Control: The neutralizer being evaluated was ten-fold serially diluted in VCM and 50 µl were added to Vero E6 cells in replicates of five for each dilution from 10^0 (neat) to 10^{-3}. Cells were scored for cytotoxicity 14 days post-inoculation.

Neutralizer Disinfectant (Cytotoxicity Control): Microbicidal active-impregnated wipes or DMEM impregnated wipes were loaded onto the Wiperator and a sterile stainless steel coupon was exposed to the wipe for 5 seconds. The exposed stainless steel coupon was placed into 1 ml of neutralizer, mixed by pipetting, and finally 10-fold serially diluted in VCM (10^0 to 10^{-3}). For each dilution, 50 µl were added to an 80% confluent Vero E6 cell monolayer in replicates of five and monitored over 14 days for CPE. Three technical replicates were used for each test wipe.

Positive Control (virus): The positive virus control was prepared by adding 10 µl of diluted low titer virus to 990 µl of VCM. The positive control was 10-fold serially diluted in VCM and 50 µl of each dilution were added to Vero E6 cells in replicates of five for each dilution from 10^0 (neat) to 10^{-3}. Cells were scored for viral CPE 14 days post-inoculation.

Neutralizer Virus Control (virus): To account for the effect of the neutralizer acting on the virus, 10 µl of low titer virus were added to 990 µl of neutralizer. The neutralizer virus control samples were 10-fold serially diluted in VCM and 50 µl were added to Vero E6 cells in replicates of five for each dilution from 10^0 (neat) to 10^{-3}. Cells were scored for viral CPE 14 days post-inoculation.

Neutralizer Disinfectant Virus Control (virus): To account for the ability for the neutralizer to affectively mitigate the effects of the disinfectant active tested, a prepared active impregnated wipe was loaded onto the Wiperator and the sterile stainless steel carrier was exposed to the active wipe for 5 seconds. The "wiped" stainless steel carrier was placed into 1 ml of neutralizer, the liquid was mixed by pipetting, and 10 µl of low-titer virus were added. The neutralization disinfectant virus control sample was incubated for 10 min at room temperature after which it was 10-fold serially diluted in VCM with

50 µl added in replicates of 5 added to Vero E6 cells for each dilution from 10^0 (neat) to 10^{-3}. Cells were scored for viral CPE 14 days post-inoculation.

Rationale for and determination of limit of detection for the Safety Plate Test

While the $TCID_{50}$ procedure is useful for calculating the titer for a virus containing solution of sufficient virus concentration (i.e., ≥1.3 log_{10}/mL), the assay fails in low virus concentration solutions where only one or two of the wells are positive in the first dilution as such is the case in efficacy trials where a microbicide is used. High lethality viruses, such as EBOV, are estimated to only require 1-10 infectious virus particles to cause infection and, as such, microbicides intended for decontaminating environmental surfaces must be able to demonstrate decontamination beyond the LOD of the $TCID_{50}$ assay. Demonstration of complete inactivation in the present study was enabled through use of the plate safety test. To determine the amount of virus needed to cause infection in a healthy Vero E6 monolayer, EBOV and VSV stocks were tenfold serially diluted to 10^0/mL (as this dilution contains, in theory, 1-10 infectious units). Serial dilutions of 10^4/mL, 10^3/mL, and 10^2/mL were used to accurately determine the amount of infectious virus in each of these dilutions. As no accurate titer could be determined for the 10^1/mL and 1^0 /mL (i.e., the dilutions we were interested in) the titers of the 10^4/mL to 10^2/mL dilutions were used to extrapolate the amounts of virus in the 10^1/mL and 1^0/mL dilutions. From the tenfold serial dilutions, 500 µL of the diluted virus were added to 6-well plates of Vero E6, incubated for 4 and 14 days for VSV and EBOV, respectively, and examined for CPE and GFP. GFP was evident after 1 day for VSV and 5 days for EBOV. It was determined that wells inoculated with 500 µL of the 10^0/mL dilution (1.36 $TCID_{50}$ units) were infected with VSV and EBOV virus. Thus, the limit of detection of the Plate Safety Test was determined to be 1.36 $TCID_{50}$ units.

Results for disinfectant neutralization effectiveness evaluation

During the evaluation of possible neutralizing agents, it was determined that 100% virus culture medium (VCM) added to the five DPW wipe dilutions prior to introduction of the EBOV/Mak or VSV in tripartite soil load (**8**) prevented inactivation of the viruses. As shown in Supplementary Table S1, VCM (neutralizer) alone and VCM + disinfectant did not cause cytotoxicity to Vero E6 cells, even applied undiluted. As shown in Supplementary Figure S2, the viral titers obtained for the virus positive controls, the neutralizer (VCM) + EBOV/Mak, and the neutralizer (VCM) + disinfectant + EBOV/Mak were indistinguishable. The disinfectant neutralizing agent that was used in each of the inactivation efficacy studies described below was VCM.

SUPPLEMENTARY TABLE S1. Cytotoxicity evaluation for Vero E6 cells exposed to negative control (VCM neutralizer) or neutralizer + DPW wipes. The lowest dilution of the post-neutralization solution which did not cause cytotoxicity to the Vero-E6 cells is shown (10^0 = undiluted)

Test Condition	Negative control (VCM)	Neutralizer + Disinfectant
Single QAC Wipes		
Replicate 1	10^0	10^0
Replicate 2	10^0	10^0

Replicate 3	10^0	10^0
AHP Wipes		
Replicate 1	10^0	10^0
Replicate 2	10^0	10^0
Replicate 3	10^0	10^0
Dual QAC Wipes		
Replicate 1	10^0	10^0
Replicate 2	10^0	10^0
Replicate 3	10^0	10^0
NaOCl Wipes		
Replicate 1	10^0	10^0
Replicate 2	10^0	10^0
Replicate 3	10^0	10^0

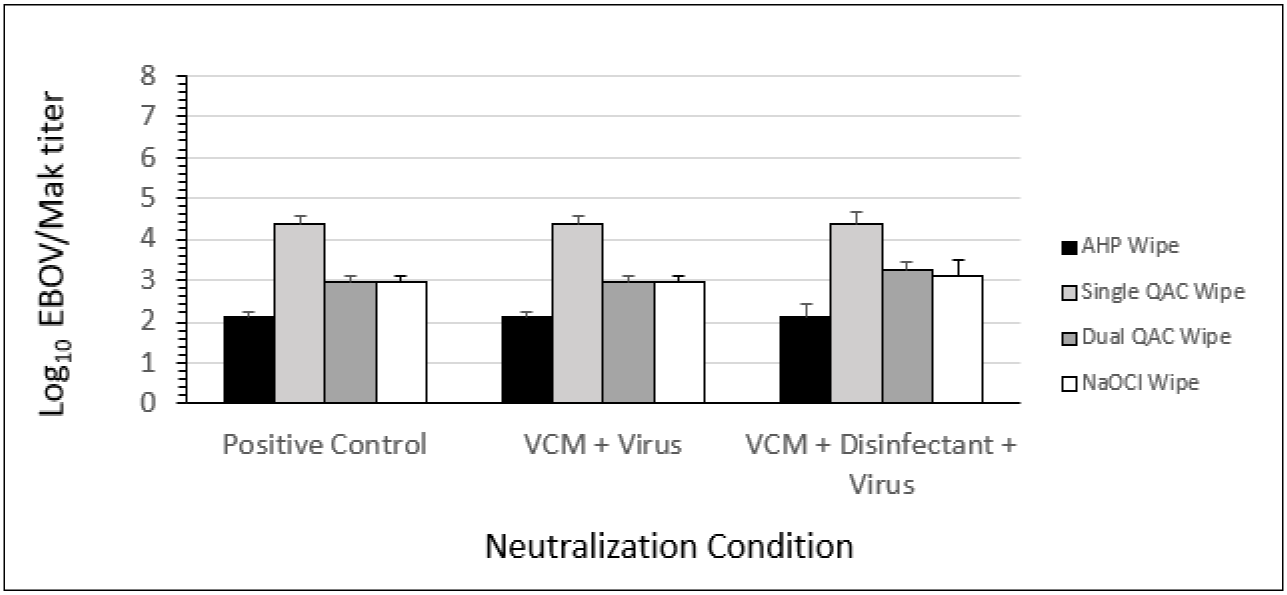

SUPPLEMENTARY FIGURE S2. Ability of VCM to neutralize the EBOV/Mak-inactivating effects of AHP, Single QAC, Dual QAC, and NaOCl disinfectant from DPW wipes.
